# Facilitating Equitable, High-Quality Cancer Screening in the Post–COVID-19 Era

**DOI:** 10.1001/jamanetworkopen.2022.15496

**Published:** 2022-06-01

**Authors:** Jennifer C. Spencer, Michael P. Pignone

**Affiliations:** Department of Population Health, Dell Medical School, University of Texas at Austin; Department of Internal Medicine, Dell Medical School, University of Texas at Austin.

By use of self-reported data from the nationally representative Behavioral Risk Factor Surveillance System, Fedewa and colleagues^1^ document the decrease in cervical and breast cancer screening during 2020, likely secondary to the direct and indirect effects of the COVID-19 pandemic. Decreases in past-year prevalence were greater for segments of the population who, because of structural inequities, were already at higher risk of not undergoing cancer screening before the pandemic and who also were the most likely to experience the adverse consequences of the pandemic (eg, those with lower educational attainment and those who identify as Hispanic).^2^ These findings raise concerns that the combined effects of the COVID-19 pandemic, both the direct risks associated with COVID-19 incidence and mortality and the indirect risks associated with deferred care for other conditions, could lead to increased all-cause mortality and worsening of health disparities.

Although these findings are alarming, 2 other aspects of the findings from Fedewa and colleagues^1^ suggest possible routes for addressing current and future inequities. First, unlike breast and cervical cancer screening, colorectal cancer (CRC) screening did not decrease significantly, as reductions in colonoscopy were counterbalanced by increased use of home-based stool testing. Moreover, the increased use of stool testing occurred disproportionately in those with lower educational attainment, suggesting that efforts to increase stool testing were reaching those at higher risk for going unscreened and, hence, may serve to reduce future inequities. An important caveat, however, is that the observed increase in stool testing must be sustained on a regular (annual or biennial) basis and be accompanied by high levels of adherence in follow-up colonoscopy after positive stool testing to reduce CRC incidence and mortality. The data available from Behavioral Risk Factor Surveillance System are not sufficient to confirm or refute that concern; however, the available data suggest a loss of adherence to stool testing over time.^3^

Second, the reductions in past-year screening observed by Fedewa and colleagues^1^ were not accompanied by similar reductions in up-to-date status, either overall or in subgroup analysis. This observation may be explained by past patterns of more frequent than recommended use of breast and cervical cancer screening compared with US Preventive Services Task Force guideline recommendations (cervical cancer screening every 3 or 5 years and mammography screening every 2 years) such that reductions in testing during 2020 did not also reduce the proportion of respondents being up to date. For CRC, the proportion who were up to date actually increased during 2020, and those groups with lower levels of up-to-date screening in previous surveys (ie, Hispanic individuals, those with lower income or educational attainment, and the uninsured) actually had larger increases than other groups, reducing but not fully closing preexisting disparities in screening. In addition, the overall levels of screening observed in 2020 remain short of the Healthy People 2030 goals: 84.3% up to date for cervical cancer screening, 74.4% for CRC screening, and 77.1% for breast cancer screening with mammography.^4^ The renewal of the Biden Cancer Moonshot initiative seeks to reduce the death rate from cancer by at least 50% over the next 25 years.^5^ Importantly, the renewal includes a focus on improving equity in cancer screening use, an investment that could help reduce existing disparities in cancer outcomes while moving the overall screening rates toward national goals.

How can these data help guide this and other investments in clinical and policy interventions? Most fundamentally, addressing uninsured status among the 10% of the population younger than 65 years would help improve insurance-related screening disparities (difference in prevalence of up-to-date screening between insured and uninsured individuals, 28.3% for mammography, 18.7% for cervical screening, and 33.5% for CRC screening),^1^ particularly if paired with efforts to improve access to primary care and, hence, a usual source of care: the gaps in screening based on having a usual source of care are similar to those seen for insured status.^1^ Many of these gaps could be most effectively addressed by expanding Medicaid in the 12 states that have not done so.^6^

In the current policy and political environment, improving access to cancer screening through federally qualified health centers (FQHCs) offers an alternative means of closing cancer screening gaps, as FQHCs serve a population at high risk of being unscreened. To date, however, FQHC screening rates have not reached the levels required to help close screening disparities, despite well-designed quality improvement efforts to do so, often because the resources available to support improvement are transitory and subject to competing demands.

One promising technique for increasing cervical and CRC screening is the use of mailed, home-based testing.^7^ The analysis by Fedewa and colleagues^1^ suggests that one reason for the positive trends observed in CRC screening was the increased use of home-based stool testing. However, we cannot tell from these data whether this increase in stool testing was driven by increased clinic-based distribution or whether mailed testing was responsible. Mailed testing offers the advantages of removing transportation barriers and frees up face-to-face visit time for other important care tasks. In the early phase of the COVID-19 pandemic, it had the advantage of not requiring an in-person visit, especially when personal protective equipment was insufficiently available. Home-based primary human papilloma virus screening has been demonstrated to be effective in research settings and in other countries but has not yet become available in the US.^8^

Importantly, for increases in screening to translate into reductions in cancer incidence and mortality, systems must also address quality of care after screening to ensure those with abnormal screening are connected with timely, appropriate follow-up for additional work-up or treatment. Gaps in appropriate follow-up are common and may be another factor associated with disparities in cancer incidence and mortality. As the data from Fedewa and colleagues^1^ show, we are making progress in implementation of effective cancer screening services, but there remains much to be done to bring the benefits of screening to the whole population.
